# Short-Segment Instrumentation with Fractured Vertebrae Augmentation by Screws and Bone Substitute for Thoracolumbar Unstable Burst Fractures

**DOI:** 10.1155/2019/4780426

**Published:** 2019-12-26

**Authors:** Jen-Chung Liao, Wen-Jer Chen

**Affiliations:** Department of Orthopedics Surgery, Bone and Joint Research Center, Chang Gung Memorial Hospital, Chang Gung University, Taoyuan, Taiwan

## Abstract

**Background:**

For thoracolumbar burst fractures, traditional four-screw (one above and one below) short-segment instrumentation is popular and has a high failure rate. Additional augmentation at the fractured vertebrae is believed to reduce surgical failure. The purpose of this study was to examine the clinical and radiographic results of patients who underwent short-segment posterior instrumentation with augmentation by screws and bone substitutes at the fractured vertebrae and to compare these data to those of patients who underwent long-segment instrumentation for thoracolumbar burst fractures.

**Methods:**

The study group had twenty patients who underwent short-segment instrumentation with additional augmentation by two screws and bone substitutes at the fractured vertebrae. The control group contained twenty-two patients who underwent eight-screw long instrumentation without vertebra augmentation. Local kyphosis and the anterior body height of the fractured vertebrae were measured. The severity of the fractured vertebrae was evaluated with the load sharing classification (LSC). Any implant failure or loss of correction >10° at the final follow-up was defined as surgical failure.

**Results:**

Both groups had similar distributions in terms of age, sex, the injured level, and the mechanism of injury before operation. During the operation, the study group had significantly less blood loss (136.0 vs. 363.6 ml, *p*=0.001) and required shorter operating times (146.8 vs. 157.5 minutes, *p*=0.112) than the control group. Immediately after surgery, the study group had better correction of the local kyphosis angle (13.4° vs. 11.9°, *p*=0.212) and restoration of the anterior height (34.7% vs. 31.0%, *p*=0.326) than the control group. At the final follow-up, no patients in the study group and only one patient in the control group experienced surgical failure.

**Conclusions:**

Patients with thoracolumbar burst fractures who received six-screw short-segment posterior fixators with augmentation at the level of the fractured vertebrae via injectable artificial bone substitute achieved satisfactory clinical and radiographic results, and this method could replace long-segment instrumentation methods used in unstable thoracolumbar burst fractures.

## 1. Introduction

Spine fractures commonly occur in the thoracolumbar region, and burst fractures account for 30% to 60% of thoracolumbar fractures [1, 2]. Severe thoracolumbar deformities and/or neurologic deficits are usually indicated for surgery. The surgical treatment of thoracolumbar burst fractures remains controversial; surgical options, including anterior surgery, posterior surgery, a combination of anterior and posterior surgery, and minimally invasive surgery, have been proposed [3, 4]. The posterior approach remains the mainstream surgical treatment for thoracolumbar burst fractures. In the last three decades, traditional four-screw short-segment posterior instrumentation has been popular, but the early implant failure rate and loss of correction rate are high. To prevent this, some surgeons have used long-segment posterior fixation. Compared to four-screw short-segment posterior fixation, long-segment posterior fixation has a better correction rate, leads to less loss of correction, and provides a better remodeling rate of the canal [5]. However, the disadvantages of long-segment posterior fixation are a long surgical time, high amounts of blood loss, and the sacrifice of two or more motion segments. More recently, six-screw short-segment posterior fixation, which involves the placement of two pedicle screws at the fractured vertebrae, has gained increasing attention because this method can reduce the surgical time and hospital costs, preserve motion segments compared to long-segment posterior fixation, and provide greater mechanical strength to prevent early implant failure compared with traditional four-screw short-segment posterior fixation [6–9]. In addition, some authors have advised that four-screw short-segment instrumentation should be augmented by bone cement or bone substitute at the fractured vertebrae for maintaining alignment and preventing implant failure [10, 11].

A finite element study by Liao et al. demonstrated that a six-screw construct with fractured body augmentation by artificial bone cement could have more stability and less stress distribution on the implant compared to the other three types of constructs (four-screw short-segment without fractured augmentation; four-screw short-segment with fractured augmentation by bone cement; and six-screw short-segment without augmentation by bone cement) for thoracolumbar burst fractures [12]. In the present study, we compared the clinical and radiographic data between patients with thoracolumbar burst fractures who underwent short-segment posterior fixation with additional augmentation by two screws and bone substitute at the fractured body and those who underwent traditional long-segment posterior fixation (two levels above and two levels below, excluding the fractured vertebrae). We hypothesized that six-screw short-segment posterior fixation with additional augmentation at the fractured vertebrae with bone substitute could limit the operation time with less blood loss and could achieve similar clinical and radiographic results as traditional long-segment posterior fixation.

## 2. Methods

This study was approved by Institutional Review Board of the Ethics Committee of our institute (number: 201901288B0). Two authors focused on this issue and began implementing these two surgical methods in 2011. To obtain at least 2 years of clinical results, data were collected from January 2011 to January 2017. All patients enrolled in this study met the following inclusion criteria: they had (A) a single-level fracture; (B) a fracture at the levels T11 to L2; (C) a type A3 or A4 burst fracture according to the AO classification [13]; (D) a fracture caused by high-energy trauma (fall from a height or motor vehicle accident); (E) an unstable burst fracture (i.e., a local kyphotic angle >20°, anterior body height collapse >50%, or spinal canal encroachment >50%) and a load sharing classification (LSC) score ≥6; (F) only posterior pedicle screw instrumentations applied without posterior, posterolateral, or interbody fusion (six-screw construct plus bone substitute inside the fractured vertebra or traditional eight-screw construct); and (G) at least 2 years of follow-up with radiographic and clinical data. These inclusion criteria were similar to those of a previous study examining the surgical outcomes of thoracolumbar burst fractures by Liao et al. [14]. In the current study, the first author performed all surgeries with short-segment instrumentation plus fractured vertebra augmentation with screws and bone substitute; the second author performed most surgeries with traditional eight-screw long instrumentation in patients. The fees associated with injectable artificial bone substitutes are not covered by National Health Insurance in our country; this artificial bone substitute was used as long as we had obtained the patient's consent. For those who agreed to use injectable artificial bone grafts, the method of reinforcement with bone grafts and screws at the fractured vertebrae was performed. The other patients underwent traditional eight-screw long instrumentation for their thoracolumbar burst fractures.

### 2.1. Radiographic Assessment

Plain radiographs were obtained before surgery, immediately after surgery, and at the final follow-up. Sagittal local kyphosis was measured from the superior endplate of the cephalic intact vertebra to the inferior endplate of the caudal intact vertebra. The normal height of the fractured vertebrae on lateral radiographs was determined by averaging the heights of the adjacent cephalic and caudal vertebrae. The percentage of the anterior height of the fractured vertebra was calculated as the anterior height of the injured vertebra/the estimated normal anterior height of the injured vertebra × 100%. Preoperative computed tomography (CT) of the spine was used to evaluate the degree of canal encroachment by the fractured fragment; the formulas adopted by Mumford et al. were used to calculate the percentage of the anterior body height and the percentage of canal compromise [15]. The severity of the fractured level was scored according to the load sharing classification using preoperative X-rays and CT scans [16]. The LSC determines the fractured body according to three components: (1) the community of the body; (2) the apposition of the fractured fragments; and (3) deformity correction after surgery. Each component is classified from one point to three points. The total LSC scores ranged from three points to nine points; more points represented greater severity of the fractured vertebrae.

### 2.2. Clinical and Neurologic Status Evaluation

The clinical results were assessed at the final follow-up visit using Denis scales [17]. The Denis scale is a five-point scale used to evaluate both pain and work status. Pain is ranked from no pain (P1) to constant and incapacitating pain requiring chronic medication (P5). Work status is ranked from return to previous labor (W1) to completely disabled (W5). Lower points on the Denis scale represented a better clinical outcome (Table 1). Preoperative and final neurological impairment were evaluated using the American Spinal Injury Association (ASIA) impairment scale.

### 2.3. Definition of Surgical Failure

The definition of surgical failure was that the implant was broken during the follow-up period or if radiographs obtained at final follow-up showed an increase of 10° or more in sagittal kyphosis compared to the local kyphosis angle measured immediately after surgery in postoperative radiographs [18].

Demographic data, including age, gender, injury level, estimated blood loss, operation time, duration of admission, time between injury and surgery, and associated injuries, were collected. All patients' surgeries were performed by these two authors; the injury grading of every patient was also confirmed by these two authors.

### 2.4. Statistics

The paired *t*-test was used to analyze differences between preoperative, postoperative, and final follow-up radiographic data within each group. The Mann–Whitney test was used to analyze numerical data between the two groups. Fisher's exact test was used for categorical variables. The level of statistical significance was set at *p* < 0.05.

## 3. Results

**Figure 1:**
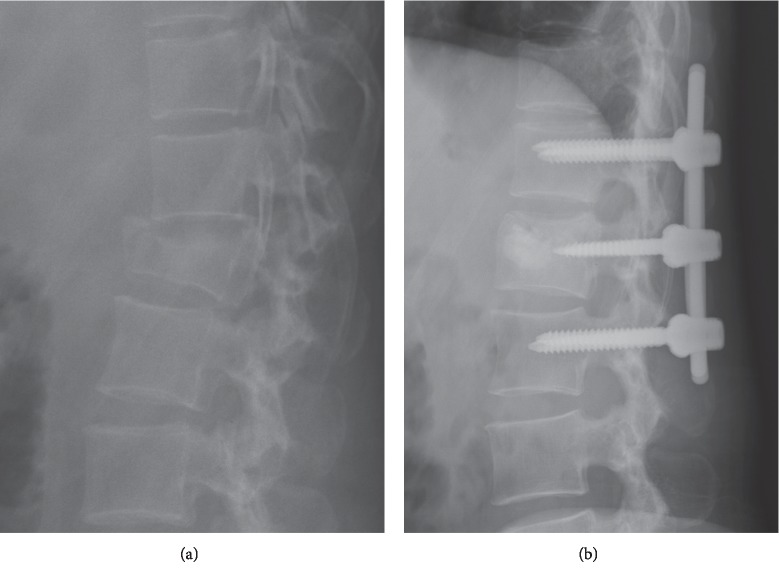
A 45-year-old female patient who underwent short-segment construct for L1 burst fracture with L1 vertebrae augmentation by injectable calcium phosphate cement and two screws (the study group). (a) Preoperative radiograph. (b) Immediately postoperative radiograph.

**Figure 2:**
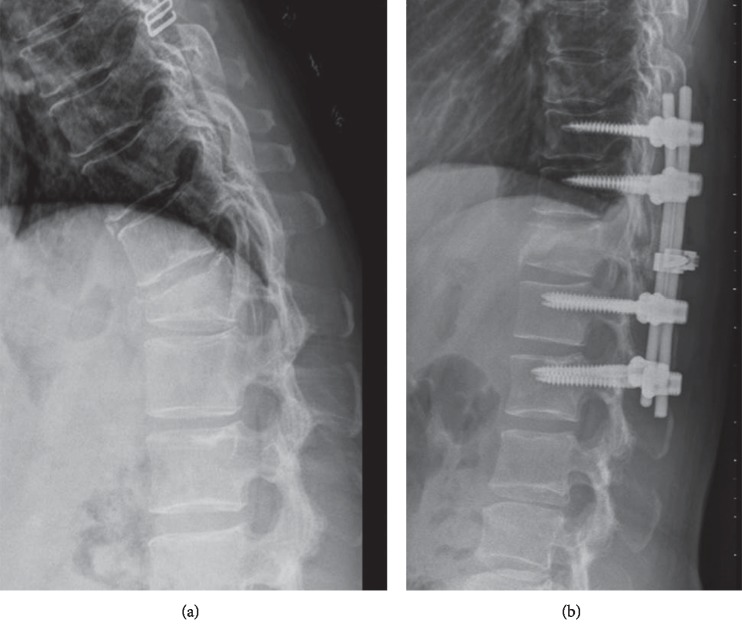
A 48-year-old female patient who underwent eight-screw long instrumentation for her T12 burst fracture (the control group). (a) Preoperative radiograph. (b) Immediately postoperative radiograph.

Forty-two patients met the inclusion criteria and were studied. Twenty patients were treated with six-screw short-segment instrumentations and bone substitute augmentation at the fractured vertebrae (the study group) (Figure 1). Eight-screw long-segment instrumentations (the control group) were performed in twenty-two patients (Figure 2). In the study group, 14 patients were type A3 and 6 patients were type A4. In the control group, 5 patients were type A3 and 7 patients were type A4. There was no statistically significant difference about patient distribution in AO classification between two groups (*p*=0.899).

No one in the study group underwent a laminectomy procedure, but three patients in the control group had received laminectomy for their progressive neurologic deficits. The total length of hospital stay in days was almost the same in the two groups. Blood loss was significantly reduced in the study group compared with that in the control group (136.0 ± 90.5 vs. 363.6 ± 306.7 ml, *p*=0.001), and a shorter operation time was observed in the study group (146.8 ± 52.0 vs. 157.5 ± 21.3 minutes, *p*=0.118). There were no statistically significant differences in sex, age, injury level, injury mechanism, or associated injuries between these two groups.  Table 2 shows the comparisons of the demographic data between these two groups.

### 3.1. Radiographic Data

In the study group, the average preoperative spinal canal encroachment as determined by CT was 52.2% ± 17.0%. The mean preoperative kyphotic angle was 20.2° ± 6.1°, which was corrected to 6.8° ± 4.6° immediately after surgery. This was a correction of 13.4° ± 5.0° (*p* < 0.001). At the final follow-up, the local sagittal angle became 9.6° ± 4.6°, and the loss of kyphosis correction was 2.9° ± 2.6°. There was still a statistically significant 10.5° correction from the time of injury to the final visit (*p* < 0.001). The mean preoperative anterior body height was 51.6% ± 10.3%, which improved to 86.3% ± 10.9% immediately after surgery (*p* < 0.001). The anterior body height was restored to 34.7% ± 11.4% with surgery. At the final follow-up, the anterior body height had collapsed significantly to 78.3% ± 12.8%. Compared to the preoperative status, the postoperative status showed that there was still a statistically significant 26.7% mean restoration at the final visit (*p* < 0.001). The mean LSC score was 6.8. In the control group, preoperative CT demonstrated that the mean spinal canal encroachment was 52.7% ± 13.9%. The average preoperative local kyphosis angle was 21.3° ± 6.9°, which was corrected to 9.4° ± 5.5° immediately after surgery. The kyphosis correction was 11.9° ± 5.8° due to the operation (*p* < 0.001). The final mean local kyphosis was 12.6° ± 6.3°. The loss of kyphosis correction was 3.2° ± 2.8°. However, there was still a statistically significant 8.7° of correction between the time of injury and the final visit (*p* < 0.001). The average preoperative anterior body height was 56.5% ± 12.8%, which improved to 85.9% ± 12.6% immediately after surgery. The postoperative anterior body height was restored by 31.0% ± 22.6%. The final anterior body height was 78.0% ± 10.7%, and the average loss of body correction was 8.0% ± 8.3%. There was still a 21.5% anterior body height acquisition between the time of injury and the final follow-up (*p* < 0.001). The mean LSC score in the control group was 6.9. The control group had a slightly higher LSC score compared with the study group, but there was no significant difference (6.9 vs. 6.8, *p*=0.628). Both groups showed no significant differences in most radiographic data. The radiographic data comparison between the two groups is demonstrated in Table 3.

### 3.2. Failure Rate

**Figure 3:**
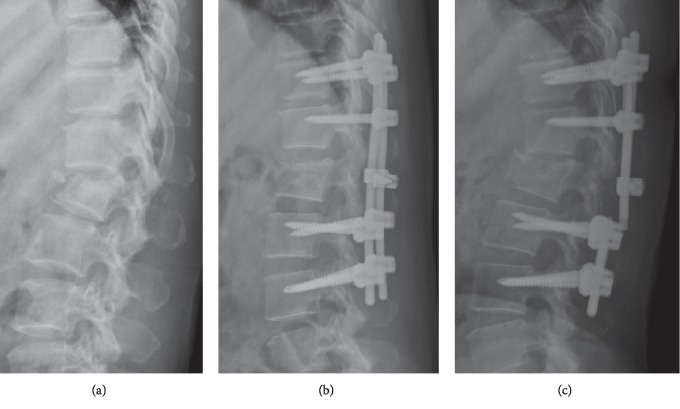
A 46-year-old female with L1 burst fracture who underwent an eight-screw long-segment instrumentation (the case with implant failure in the control group). (a) Preoperative radiograph. (b) Immediately postoperative radiograph. (c) The final radiograph showed the rods were broken at L1-2 region.

In the study group, the failure rate was 0% (0/20). In the control group, only one patient met the criteria for failure and the failure rate was 4.5% (1/22) (Figure 3). The LSC score of this patient was 7, but this patient underwent laminectomy for her progressively neurologic deficit status (ASIA C).

### 3.3. Clinical Data

In the study group, the mean pain score was 1.25 ± 0.55, and the mean work score was ultimately 1.50 ± 0.69. In the control group, the average pain score and work score were 1.55 ± 0.74 and 1.82 ± 1.29, respectively. No statistically significant differences in pain or work scores (*p* = 0.102 and 0.731, respectively) were observed between the two groups (Table 4).

### 3.4. Neurologic Status

According to the ASIA grading system, one, five, and 14 patients in the study group were classified as grades C, D, and E, respectively; and one, one, one, two, and 17 patients in the control group were classified as grades A, B, C, D, and E, respectively, before surgery. There was no significant difference in the distribution of neurologic deficits between the two groups (30% vs. 23%, *p*=0.480). Patients from both groups who had a preoperative neurologic deficit could obtain some degree of neurologic recovery through surgery, and no one had sustained neurologic deterioration due to surgery (Table 5).

## 4. Discussion

Although whether anterior or posterior surgery is the most effective treatment for burst fractures is still under debate, posterior indirect decompression and fixation has become popular in the last three decades, especially since the development of pedicle screw instrumentation. It remains controversial as to whether short-segment instrumentation or long-segment instrumentation is the optimal treatment for thoracolumbar burst fractures [19, 20]. Posterior short-segment fixation is associated with a high rate of early implant failure [21]. A theory has been proposed that a large defect in the fractured vertebrae is created during posterior instrumentation after the application of distraction force by short-segment instrumentation, which leads to early implant failure and/or local re-kyphosis [17]. Therefore, augmentation techniques were designed and used to prevent this complication. Transpedicular grafting with autogenous bone grafts or artificial bone from the injured anterior body in addition to short-segment fixation has been suggested as a possible solution by Alanay et al. and Liao et al. [14, 22]. Adding two screws to the fractured vertebrae for augmentation is another option. The advantages of this method include providing a mass effect, preventing the vertebrae from collapse, and supporting the anterior column to enhance the stability of the construct. Recent literature has shown that the six-screw short-segment construct can effectively prevent early implantation, especially in those with LCS ≤7, and can provide satisfactory clinical results for thoracolumbar burst fractures [23, 24]. Compared to traditional four-screw short-segment fixation, short-segment fixation with screws placed at the fractured level can achieve better correction with a lower rate of implant failure [7, 8]. Lin et al. and Liao et al. also demonstrated that a six-screw short-segment construct had a lower implant failure rate with better alignment maintenance compared to transpedicular grafting with short-segment instrumentation [18, 25].

Tezeren and Kuru studied 18 consecutive patients with thoracolumbar burst fractures involving nine patients treated with four-screw short-segment pedicle fixation and nine patients treated with long-segment instrumentation; the final outcome regarding local kyphosis and anterior body compression was better with long-segment instrumentation than with four-screw short-segment pedicle fixation [26]. Guven et al. studied 72 patients with thoracolumbar burst fractures and found that the use of screws at the fracture level could provide better kyphosis correction and could lower the implant failure rate with both short-segment instrumentation and long-segment instrumentation; the radiographic data from the six-screw short-segment fixation were similar to the data from the eight-screw long-segment instrumentation [6]. In our experience, early implant failure of six-screw short-segment instrumentation in thoracolumbar burst fracture is still seen (Supplement Materials ([Supplementary-material supplementary-material-1])). That is why we used long-segment instrumentation or short-segment instrumentation with fractured vertebrae augmentation by combination of intermediate screws and bone graft for thoracolumbar burst fractures. In the present study, the immediate postoperative local kyphosis level and kyphosis correction in the study group were superior to those in the control group (6.8° vs. 9.4° and 13.4° vs. 11.9°, respectively), although there were no significant differences; this result indicates that the placement of the pedicle screw at the fracture level provides a mass effect to the buttress vertebra endplate and corrects local kyphosis via the screw's bending force, similar to a rod sleeve effect [27, 28]. In addition, the failure rate and loss of correction at the final follow-up were lower in the study group than in the control group. The preoperative LSC score was similar in both groups (6.8 vs. 6.9, *p*=0.628), which suggests that patients in both groups sustained similarly comminuted vertebrae that the construct should bear. The above results indicate that the stability of the construct in the study group might be equal to or stronger than that in the control group. A finite element study on thoracolumbar burst fractures demonstrated that the stability of the six-screw construct could be enhanced by applying bone grafts inside the fractured vertebrae with less stress distribution on the implant compared to a six-screw construct without fractured vertebra augmentation [12].

Although without statistically significant difference, the current study demonstrated that a longer operation time was observed in the control group than in the study group (146.8 vs. 157.5 minutes, *p*=0.112), which was similar as shown in previous literatures [6, 26]. Dramatic differences in blood loss between the groups (136.0 cc. vs. 363.6 cc, *p*=0.001) were observed in this study, which we believe were caused by the fact that there were more patients in the control group with progressive neurologic deficits, and a higher number of posterior decompression procedures were needed, which led to greater blood loss. According to Denis's pain and work scale, the two groups showed similar clinical results without significant differences. However, we found that patients in the control group had slightly higher pain (1.55 ± 0.74 vs. 1.25 ± 0.55) and work (1.82 ± 1.29 vs. 1.50 ± 0.69) scores than those in the study group, which was also related to the fact that there were more patients with neurologic deficits in the control group at the final follow-up.

In this study, we included all patients with an AO classification type A3/A4 burst fracture who underwent posterior instrumented surgeries. The AO classification replaced the three-column model-based Denis classification in our institute on evaluating thoracolumbar fractures because the AO classification can identify a wide array of fractures including more than 50 subtypes using the 3-3-3 AO principle. However, some literature has reported that the complexity of the AO classification limits its routine clinical practice due to its fair interobserver reliability and moderate intraobserver reliability [29, 30]. In 2005, the thoracolumbar injury severity score (TLISS) was introduced to evaluate thoracolumbar fractures based on three injury characteristics: (1) the mechanism of injury, (2) the integrity of the posterior ligament complex (PLC), and (3) the neurologic status of patients [31]. Disruption of PLC damages the stability of spinal column, which is an indication for surgical intervention. However, Rihn et al. suggested that the evaluation of PLC injury suffered from poor reliability and reproducibility, even with a magnetic resonance imaging (MRI) examination [32]. Furthermore, in patients who have undergone CT examination, they cannot routinely undergo MRI for their spinal injury according to our National Health Insurance regulations. We feel it is not easy to evaluate posterior complex injury preoperatively, which is why we did not use TLICS in this study.

Indeed, our study still had some limitations. One was that this study was reviewed retrospectively with a relatively small case number. Another was that three cases in the control group and none in the study group received laminectomy procedure, which might be a bias for the study.

## 5. Conclusions

In conclusion, the data showed that in combination with bone substitute augmentation at the fractured level, short-segment instrumentation could achieve similar clinical and radiographic results as long-segment instrumentation for thoracolumbar burst fractures. Further, this approach also reduced blood loss and operation time. A six-screw short-segment construct with augmentation at the fractured vertebrae by bone substitute is adequate for unstable thoracolumbar burst fractures with an average LSC score of 7.

## Figures and Tables

**Table 1:** Denis pain scale and work scale.

Pain scale
P1: no pain
P2: occasional pain not requiring medication
P3: moderate pain requiring occasional medication
P4: moderate to severe pain requiring frequent medication
P5: constant incapacitating pain requiring chronic medication
Work scale
W1: returned to previous employment
W2: capable but did not return to previous employment
W3: unable to return to previous employment and currently employed in a different full-time job
W4: unable to return to previous employment and currently working part-time or frequently absent from work because of pain
W5: completely disabled and unable to work

Source: Denis F et al. Clin Orthop Relat Res. 1984; 189 : 142-9. P = pain; W = work.

**Table 2:** Patient demographic data.

Characteristic	Study	Control	*p* values
	(*N* = 20)	(*N* = 22)	
Age (years)	41.6 ± 12.7	42.9 ± 10.6	0.811
Gender			
Female	7	9	0.615
Male	13	13	
Level			
T11	0	0	0.648
T12	4	7	
L1	12	12	
L2	4	3	
Hospital stay (days)	12.1 ± 5.7	11.0 ± 6.1	0.253
Injury to operation interval (days)	4.2 ± 2.2	4.4 ± 4.2	0.415
Operation time (min)	146.8 ± 52.0	157.5 ± 21.3	0.118
Blood loss (c.c.)	136.0 ± 90.5	363.6 ± 306.7	0.001
Mechanism			
Fall	18	16	0.257
MVA	2	6	
Associated injury			
Yes	8	5	0.191
No	12	17	

MVA = motor vehicle accident.

**Table 3:** Radiographic data of surgery.

Parameter	Study (*N* = 20)	Control (*N* = 22)	*p* values
Failure rate	0/20 (0%)	1/22 (4.5%)	1.000
Preoperative canal encroachment (%)	52.2 ± 17.0	52.7 ± 13.9	0.772
Local kyphosis (degree)			
Preoperative	20.2 ± 6.1	21.3 ± 6.9	0.529
Postoperative	6.8 ± 4.6	9.4 ± 5.5	0.170
Final	9.6 ± 4.6	12.6 ± 6.3	0.107
Correction by surgery	13.4 ± 5.0	11.9 ± 5.8	0.212
Loss of correction at final	2.9 ± 2.6	3.2 ± 2.8	0.821
Preoperative vs. Postoperative	*p* < 0.001	*p* < 0.001	
Postoperative vs. Final	*p* < 0.001	*p* < 0.001	
Preoperative vs. Final	*p* < 0.001	*p* < 0.001	
Anterior body height (%)			
Preoperative	51.6 ± 10.3	56.5 ± 12.8	0.092
Postoperative	86.3 ± 10.9	85.9 ± 12.6	0.669
Final	78.3 ± 12.8	78.0 ± 10.7	0.960
Correction by surgery	34.7 ± 11.4	31.0 ± 22.6	0.326
Loss of correction at final	8.0 ± 5.4	8.0 ± 8.3	0.279
Preoperaitve vs. Postoperative	*p* < 0.001	*p* < 0.001	
Postoperative vs. Final	*p* < 0.001	*p* < 0.001	
Preoperaitve vs. Final	*p* < 0.001	*p* < 0.001	
Load sharing score			
6	7	7	0.628
7	10	9	
8	3	5	
9	0	1	

**Table 4:** Clinical outcomes using Denis scale.

Parameter	Study	Control	*p* values
	(*N* = 20)	(*N* = 22)	
Pain scale			
1	16	12	0.102
2	3	9	
3	1	0	
4	0	1	
5	0	0	
Work scale			
1	12	13	0.731
2	6	5	
3	2	1	
4	0	1	
5	0	2	

**Table 5:** Distribution of neurologic status according ASIA impairment scale.

	A	B	C	D	E
Study (*N* = 20)					
Preoperative	0	0	1	5	14
Final	0	0	0	1	19
Control (*N* = 22)					
Preoperative	1	1	1	2	17
Final	1	0	1	1	19

ASIA = American Spinal Injury Association.

## Data Availability

The data used to support the finding of this study are available from the corresponding author upon request.

## Supplementary Materials

Supplementary Materials An early implant failure (see arrow) could happen in a patient with thoracolumbar burst fracture who underwent six-screw short-segment instrumentation.
