# Supplementary figures and images for: Short-Segment Instrumentation with Fractured Vertebrae Augmentation by Screws and Bone Substitute for Thoracolumbar Unstable Burst Fractures
Source: Biomed Res Int. 2019 Dec 26;2019:4780426. doi: 10.1155/2019/4780426 (PMC6948339; doi:10.1155/2019/4780426)

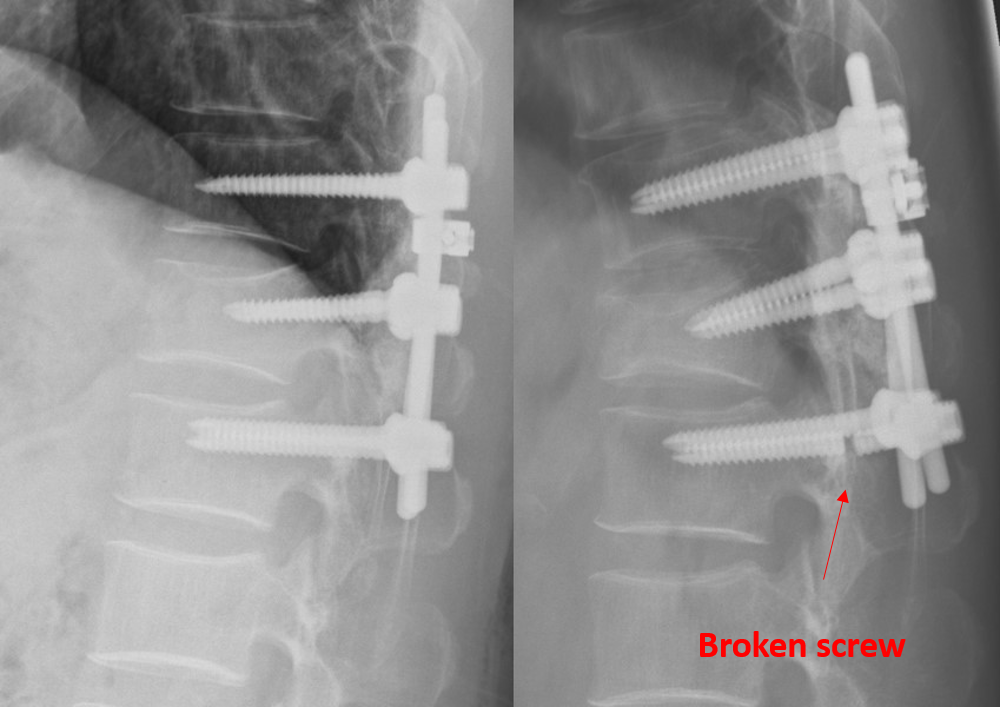

Supplement: Supplementary Materials — An early implant failure (see arrow) could happen in a patient with thoracolumbar burst fracture who underwent six-screw short-segment instrumentation. [file 4780426.f1.tif]
